# Absorptive capacity, technological innovation, and product life cycle: a system dynamics model

**DOI:** 10.1186/s40064-016-3328-5

**Published:** 2016-09-26

**Authors:** Bo Zou, Feng Guo, Jinyu Guo

**Affiliations:** School of Management, Harbin Institute of Technology, 92 West Dazhi Street, Nan Gang District, Harbin, 150001 China

**Keywords:** Absorptive capacity, Technological innovation, Product life cycle, System dynamics

## Abstract

**Background:**

While past research has recognized the importance of the dynamic nature of absorptive capacity, there is limited knowledge on how to generate a fair and comprehensive analytical framework. Based on interviews with 24 Chinese firms, this study develops a system-dynamics model that incorporates an important feedback loop among absorptive capacity, technological innovation, and product life cycle (PLC).

**Results:**

The simulation results reveal that (1) PLC affects the dynamic process of absorptive capacity; (2) the absorptive capacity of a firm peaks in the growth stage of PLC, and (3) the market demand at different PLC stages is the main driving force in firms’ technological innovations. This study also explores a sensitivity simulation using the variables of (1) time spent in founding an external knowledge network, (2) research and development period, and (3) knowledge diversity. The sensitivity simulation results show that the changes of these three variables have a greater impact on absorptive capacity and technological innovation during growth and maturity stages than in the introduction and declining stages of PLC.

**Conclusions:**

We provide suggestions on how firms can adjust management policies to improve their absorptive capacity and technological innovation performance during different PLC stages.

## Introduction

Absorptive capacity is regarded as important for firm innovation and performance and has been a hot topic in the field of strategy and organization for more than two decades (Barta et al. 2016; Huang et al. 2015; Sopelana et al. 2014; Vasudeva and Anand 2011). The dynamic nature of absorptive capacity is one of the major research areas in this field (Cohen and Levinthal 1990; Mäkinen and Vilkko 2014; Todorova and Durisin 2007; Zahra and George 2002), and two main research streams exist. The first is the dynamic process of absorptive capacity, as described by Cohen and Levinthal (1990), whose research mainly focuses on three dimensions—recognition, assimilation, and exploitation. Followers of this concept suggested four dimensions (acquisition, assimilation, transformation, and exploitation) (Zahra and George 2002), three process dimensions (exploratory learning, transformative learning, and exploitative learning) (Lane et al. 2006), and five dynamic process dimensions (recognition, acquisition, assimilation or transformation, and exploitation) (Todorova and Durisin 2007). The second research stream attempted to explore the coevolution between absorptive capacity and the external environment. For example, Van Den Bosch et al. (1999) stress that the features of a firm’s absorptive capacity were related to the knowledge property that the firm has in its environment. In that vein, Mäkinen and Vilkko (2014) trace the evolution of absorptive capacity within a turbulent competitive industry environment.

Although previous studies have showed the dynamic nature of absorptive capacity, much remains to be explored on the subject. First, past research has been primarily concerned with the dynamic process of absorptive capacity or its coevolution with the external environment (Cohen and Levinthal 1990; Lane et al. 2006; Mäkinen and Vilkko 2014; Todorova and Durisin 2007; Van Den Bosch et al. 1999; Zahra and George 2002) and much less attention has been focused on an integrative view. Second, relative to qualitative research, quantitative research in regards to absorptive capacity is inadequate. As Todorova and Durisin (2007) report, the study of absorptive capacity requires using longitudinal research methods and process models, which will help to reveal the dynamic evolution process. Thus, it is important to investigate the dynamic coevolution of absorptive capacity process with the external environment by applying quantitative research methods. For example, the external environment affects and evolves with absorptive capacity (Cohen and Levinthal 1990; Zahra and George 2002), which may influence the different processes of absorptive capacity. Moreover, quantitative data may enhance the understanding of the dynamic evolution of absorptive capacity (Mäkinen and Vilkko 2014; Todorova and Durisin 2007).

In order to fill this research gap, this paper aims to explore the dynamic coevolution of the absorptive capacity with external environment. Todorova and Durisin (2007) indicate that applying the quantitative research methods of system dynamics provides benefits in revealing the dynamic nature of absorptive capacity. System dynamics is a kind of simulation method that is suitable for modeling complex systems and involves interactions and various types of feedback (Sterman 2000; Todorova and Durisin 2007). Product life cycle (PLC) is a type of environment (Hambrick 1983) that may be considered as an external environment. This paper constructs a dynamic model of absorptive capacity that includes three subsystems (external knowledge sources, knowledge storage, and technology-innovation achievements) involving the dynamic process of absorptive capacity coevolution with different PLC stages.

In addition to filling a research gap, this paper also seeks to make contributions. First, we build a system model that considers both the dynamic process of absorptive capacity as well as one kind of external environment—the product life cycle. Secondly, a sensitivity analysis of time spent in funding an external knowledge network, research and development (R&D) period, and knowledge diversity enriches our understanding of absorptive capacity and technological innovation. Finally, this paper attempts to build a system-dynamics model of absorptive capacity, which provides a platform for further study.

## Background

### Absorptive capacity

Over the past 20 years, studies within the strategic management literature have cited the importance of absorptive capacity as a way to achieve better firm performance (Mäkinen and Vilkko 2014; Wales et al. 2013). The concept of absorptive capacity was put forward and applied to the firm level by Cohen and Levinthal (1990). Although many definitions of absorptive capability exist in the literature, the concepts from Cohen and Levinthal (1990) and Zahra and George (2002) have profoundly influenced the development of absorptive capacity theory. Cohen and Levinthal (1990) define absorptive capacity as “the ability of a firm to recognize the value of new, external information, assimilate it, and apply it to commercial ends” (Cohen and Levinthal 1990, p. 128). According to Zahra and George (2002), absorptive capacity was viewed as “a set of organizational routines and processes by which firms acquire, assimilate, transform, and exploit knowledge to produce a dynamic organizational capability*”*(Zahra and George 2002, p. 186). One important contribution of Zahra and George’s work is the distinction between potential and realized absorptive capacity. According to their point of view, potential absorptive capacity concentrated on knowledge acquisition and assimilation, but realized absorptive capacity contained knowledge transportation and exploitation (Zahra and George 2002).

Based on the notion of absorptive capacity proposed by Cohen and Levinthal (1990) and Zahra and George (2002), this paper develops a basic model for exploring the dynamic nature of absorptive capacity. The model has three aspects. First, it integrates the abovementioned views and defines absorptive capacity as five process dimensions—valuing, acquisition, assimilation, transformation, and exploitation (Cohen and Levinthal 1990; Zahra and George 2002). Second, the model is divided into three subsystems: external knowledge source, knowledge storage, and technology innovation achievements. The external knowledge source subsystem is the system of developing an external knowledge network by valuing and identifying relative knowledge based on current market demand and a firms’ innovation situation. The knowledge storage subsystem is the system that forms a firm’s knowledge storage by assimilating external knowledge. The technology innovation achievements subsystem is a system that transforms and exploits a firm’s knowledge storage, thus achieving technology innovations. Third, dynamic feedback loops exist among the three subsystems, and the five process dimensions are used to connect them and generate the system as a whole.

### Product life cycle

Since the idea of product life cycle (PLC) was introduced several decades ago, it has attracted widely attention as well as a great deal of research (Anderson and Zeithaml 1984; Mahapatra et al. 2012; Rink and Swan 1979; Sang 2016). Östlin et al. (2009) describe the concept of PLC as “the evolution of a product, measured by its sales over time” (Östlin et al. 2009, p. 1000). PLC denotes the life cycle stage of a firm’s focal product, characterizes the product-market context, and represents a well-recognized external contingent factor that explains a firm’s strategy (Chen et al. 2016; Mahapatra et al. 2012; Thietart and Vivas 1984).

In general, every product passes through four stages: introduction, growth, maturity, and decline (Anderson and Zeithaml 1984; Barsila et al. 2015; Golder and Tellis 2004; Rink and Swan 1979). Each stage is defined by a turning point in the growth rate of sales (Huang and Tzeng 2008). In the introduction stage, new products are usually developed based on an observed need and a market demand. Although the market demand is initially low, it slowly rises. In the growth stage, the market demand increases rapidly, and competitors enter the market with their own products while in the meantime, the product is continuously improved. In the maturity stage, the total demand begins to level off, although it may continue to grow in some areas and decline in others. Decline is the period in which sales decrease persistently until the product disappears.

Many coevolutionary studies suggest that absorptive capacity enables or restricts the level and range of exploration adaptations and should be related to environment (Cohen and Levinthal 1990, 1994, 1997; Lewin et al. 1999; Wales et al. 2013). For instance, Van Den Bosch et al. (1999) studies the coevolution of a firm’s path-dependent absorptive capacity and the knowledge environment. Wales et al. (2013) indicates that environment dynamism and hostility have been shown to influence absorptive capacity. Studies in marketing and strategy management have reported that PLC is the fundamental variable that influences business strategy and performance (Anderson and Zeithaml 1984; Chen et al. 2016; Mahapatra et al. 2012). At different PLC stages, demand will influence a firm’s absorptive capacity and therefore its technological innovation performance. Absorptive capacity can enable a firm to change to match the dynamic market (Cohen and Levinthal 1990; Zahra and George 2002). In this study, PLC is regarded as an important external variable that affects absorptive capacity and technological innovation dynamically.

## Research model and methods

### Conceptual model

This study adopts the method of system dynamics to reveal the dynamic effect relationship between absorptive capacity, technological innovation, and product life cycle (PLC). The fundamental principle of system dynamics is that the structure of the system gives rise to its behavior (Sterman 2001). Before revealing the behavior of a system, the structure of the system should first be described. Therefore, we describe the overall model of the relationship between absorptive capacity, technological innovation, and PLC based on a theoretical background and the interviews.

In theory, according to the research of Cohen and Levinthal (1990) and Zahra and George (2002), as absorptive capacity contains five process dimensions—valuing, acquisition, assimilation, transformation, and exploitation—we divide the relationship between absorptive capacity and technological innovation into three subsystems: external knowledge source, knowledge storage, and technology-innovation achievements. In addition, because PLC is an important context variable influencing business strategy and performance (Anderson and Zeithaml 1984; Chen et al. 2016; Mahapatra et al. 2012), it is regarded as an important external variable that influences firms’ absorptive capacity and technology innovation.

Through our investigation and interviews with 24 firms in the industries of IT, manufacturing, and pharmacy, we find that despite the different product life cycles in different sectors (the product life cycle in IT, manufacturing, and pharmacy is nondurable, medium, and durable respectively), all have a high degree of similarity in the internal mechanism of the relationship between absorptive capacity and technological innovation. Generally speaking, this similarity is mainly reflected in the interaction between the sub-system of the external knowledge source, knowledge storage, and technology innovation achievements. Our theoretical background and interview results guarantee the validity of the conceptual model constructed in this study.

**Fig. 1:**
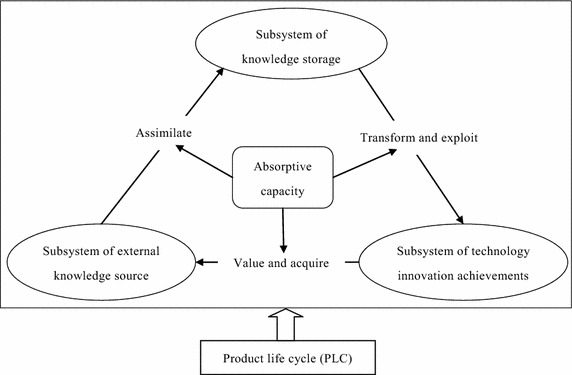
Conceptual model

It is worth emphasizing that although we consider the PLC, this study is a firm-level study; that is, we consider the PLC as an external variable rather than endogenous variable that affects the evolution of absorptive capacity and technology innovation. The conceptual model can be seen in Fig. 1.

As shown in Fig. 1, the whole system is divided into three subsystems, with the following primary relationships. First, a firm values and acquires external knowledge in accordance with current market demand and its own innovation situation. This is the “value” and “acquire” arrow from the technology-innovation-achievements subsystem to the external-knowledge-source subsystem. Second, the firm develops processes, policies, and procedures to assimilate the knowledge internally, as shown by the “assimilate” arrow from the external-knowledge-source subsystem to the knowledge-storage subsystem. Third, the firm transforms and utilizes the new knowledge to create new products, which is shown by the “transform” and “exploit” arrow from the knowledge-storage subsystem to the technology-innovation-achievements subsystem. It should be emphasized that the relationships among the three subsystems are not static, but rather form a dynamic feedback process.

To further explore the firm’s absorptive capacity and technological innovation in different market stages, this paper introduces the PLC as an important external variable through which to study the market demand and conditions. It also examines the behavior and performance of absorptive capacity and technological innovation at different PLC stages. In sum, our model considers the dynamic process of absorptive capacity and the dynamic impact of PLC on absorptive capacity.

### System-dynamics modeling

System dynamics can be used to deal with sophisticated policy issues and social problems. It also provides us an approach to test the framework of a dynamic and complex system that has various interactions among variables over a period of time (Bérard and Perez 2014; Cui et al. 2011b). System dynamics can also serve as a laboratory for (1) addressing social problems by implementing and testing assumptions that, in practice, are impossible to try ahead of time, and (2) adjusting and integrating these assumptions in a more logical and testable way for the next phase (Homer 1996; Sterman 2001). A basic principle of system dynamics is that a system’s behavior is determined by its structure (Bérard and Perez 2014; Sterman 2001). Applying the tools and insights from system dynamics helps us to better understand the evolution and effects of absorptive capacity, which are dynamic and complex (Todorova and Durisin 2007). Therefore, the study builds a system-dynamics model based on earlier modeling efforts and empirical evidence on absorptive capacity and technological innovation.

### Field interviews

We collected data from field interviews conducted with 24 Chinese firms, including 8 manufacturing companies, 12 information technology firms, and 4 pharmaceutical firms. We chose these three industries because the PLC in IT, manufacturing, and pharmacy is nondurable, medium, and durable, respectively. Through these interviews, we hoped to draw conclusions for general value (Rink and Swan 1979).

The interviewees in this research were firm executives, middle managers, principal officers in charge of technical work, and front-line technical staff. Five to eight people (including the interviewer) comprised each interview group, and the interviews were held mostly in the form of group discussions aimed at encouraging “brainstorming” and reaching a wide range of agreement. The interview questions were pre-designed as open and semi-structured questions.

From these interviews, we obtained clues about feedback loops among different variables in the system as well as detailed parameters of the system as a whole, which included several variables (the parameters are discussed in the Table 2 of “Appendix”). Finally, based on these feedback loops and parameters, we created a general sketch of the integral system and laid a foundation for developing the system-dynamics model.

### Model

**Fig. 2:**
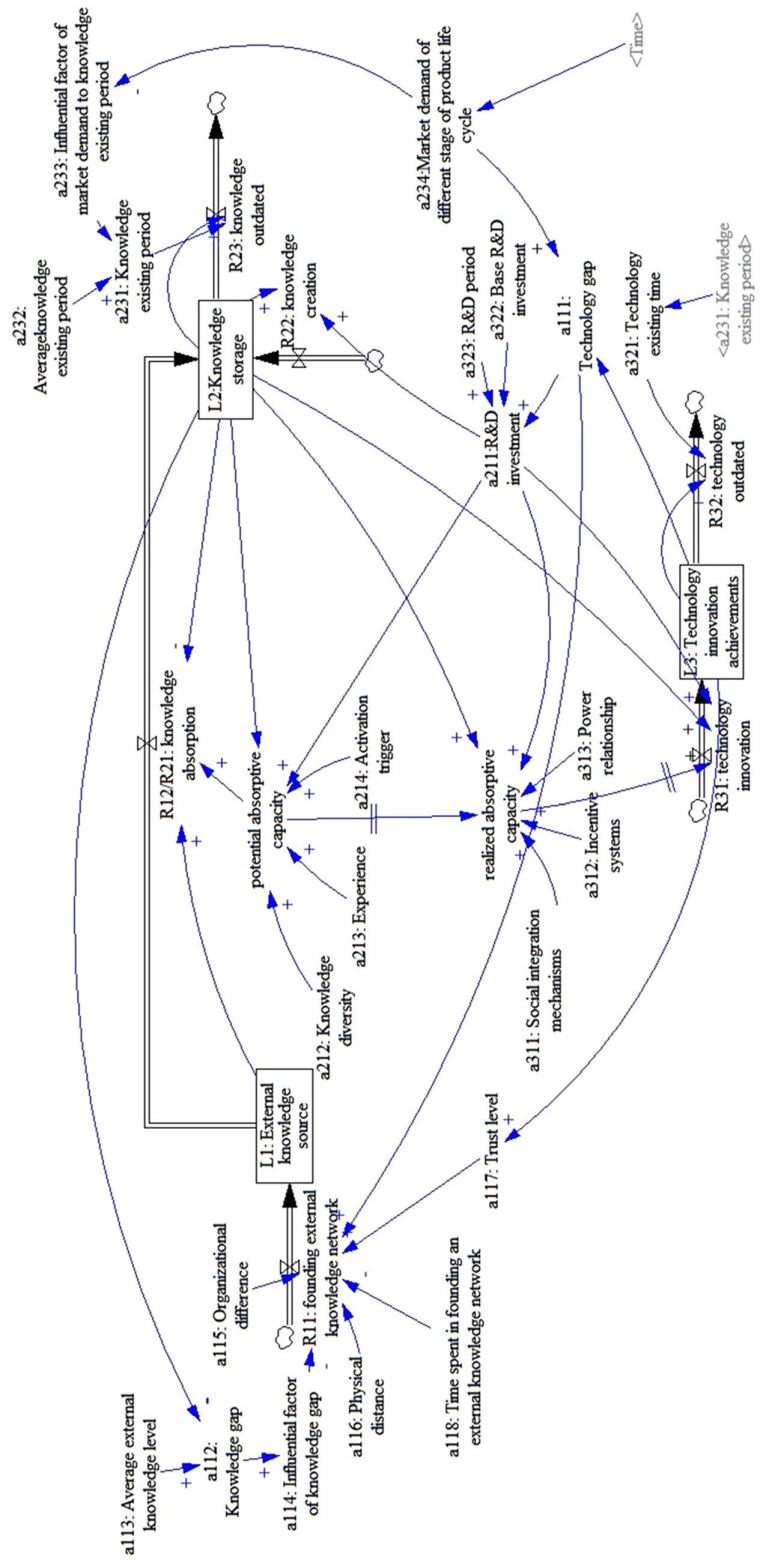
The stock and flow diagram of the whole system

 Figure 2 shows a firm based on the current market demand and its own innovation situation in the context of PLC values and acquires external knowledge by developing external knowledge sources. The firm then develops processes, policies, and procedures to assimilate the knowledge internally and form the knowledge storage. Finally, the firm transforms and utilizes the new knowledge to achieve the firms’ technology innovation. Further elaboration on the system-dynamics model is shown in Fig. 2.

#### External-knowledge-sources subsystem

Part of a firm’s knowledge is from external sources, which are key to innovation (Barta et al. 2016; Park 2014). The external knowledge source (L_1_) of a firm is influenced by the inflow rate of establishing external knowledge (R_11_) and the outflow rate of external knowledge source (R_12_), with the initial value being zero:

$$\left\{ {\begin{array}{*{20}l} {\frac{{{\text{d}}L_{1} (t)}}{{{\text{d}}t}} = R_{11} \left( t \right) - R_{12} (t)} \hfill \\ {L_{1} (0) = 0} \hfill \\ \end{array} } \right..$$

The technology gap (a_111_) is the original driving force of the inflow rate to establish the external knowledge (R_11_) and it is an independent factor of R_11_. In this research, a_111_ refers to the gap between a firm’s current technology situation and the technology demanded by the market. When the value becomes larger, the firm will be stimulated to look for more external knowledge sources in order to find ways to improve its technology.

The knowledge gap (a_112_) between the firm and its external link has an uncertain influence on R_11_. Research results have shown that the relationship between the knowledge gap and knowledge acquisition is an inverted U-shape (Schildt et al. 2012). In this study, the knowledge gap is the normalized difference between knowledge storage (L_2_) and the average external knowledge level (a_113_):

$$a_{112} \frac{{|a_{113} - L_{2} |}}{{a_{113} }}.$$

The relationship between the knowledge gap and knowledge acquisition is simulated as a factor that has multiple influences on the knowledge gap (a_114_), which can be represented in the form of a table function:

$$a_{114} = {\text{with-lookup(a}}_{ 1 1 2} ).$$

In addition, other variables influence the inflow rate of establishing external knowledge (R_11_). These variables include organizational difference (Lane and Lubatkin 1998), physical distance (Galbraith 1990), and trust level between a firm and its external link (Yli-Renko et al. 2001). Hence, the negative organizational difference (a_115_), the negative physical distance (a_116_), and the positive trust (a_117_) are all modeled as dimensionless parameters that are assembled according to different weights into one combined factor based on the interview; therefore,

$$R_{11} (t) = a_{111} \times a_{114} \times (0.3/a_{115} + 0.2/a_{116} + 0.5 \times a_{117} )/a_{118} .$$

Because the outflow rate of the external knowledge source (R_12_) is also the inflow rate of the knowledge storage (R_21_), it will be discussed as a part of the knowledge-storage subsystem.

**Fig. 3:**
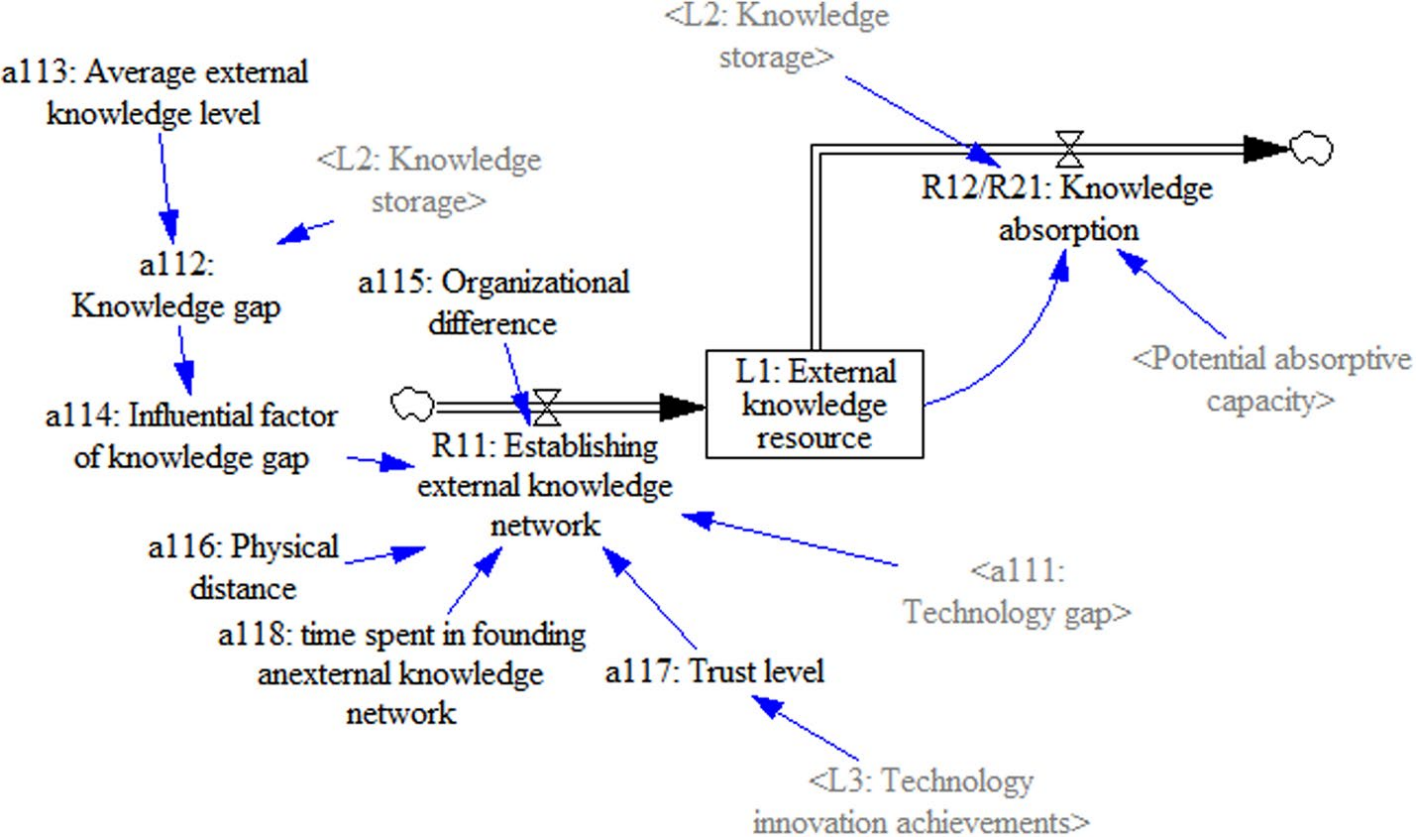
Separate stock-flow model of external knowledge source subsystem

Figure 3 shows the stock-flow diagram of this subsystem.

#### Knowledge-storage subsystem

Knowledge storage refers to the amount of knowledge elements that a firm has piled up, which affects a firm’s R&D activities and potential ability to achieve technology innovation (Sciascia et al. 2014; Wanzenböck et al. 2013); i.e., the more knowledge storage a firm has, the more technology-innovation achievements the firm is likely to achieve.

A firm’s knowledge storage (L_2_) is influenced by the inflow rate of knowledge absorption (R_21_), knowledge creation (R_22_), and the outflow rate of outdated knowledge (R_23_), with a specific initial value. The formulation of the knowledge storage (L_2_) is:

$$\left\{ {\begin{array}{*{20}l} {\frac{{{\text{d}}L_{2} (t)}}{{{\text{d}}t}} = R_{21} (t) + R_{22} (t) - R_{23} (t)} \hfill \\ {L_{2} (0) = {\text{Ini}}\_L_{2} } \hfill \\ \end{array} } \right..$$

According the results of our interviews, knowledge absorption (R_21_) is impacted by the rate of the external knowledge source (L_1_) and the knowledge storage (L2). Potential absorptive capacity (PACAP) makes it easier for a firm to acquire and assimilate external knowledge (Lane and Lubatkin 1998; Zahra and George 2002) and therefore it is the key factor that influences knowledge absorption; hence,

$$R_{21} (t) = PACAP \times L_{1} /L_{2} .$$

The change of PACAP is influenced by multiple factors, including R&D investment (a_211_), knowledge diversity (a_212_), experience (a_213_), and activation trigger (a_214_), which are positively related to PACAP (Todorova and Durisin 2007; Zahra and George 2002). According to our field interview results, 20 % of R&D investment (a_211_) is applied to building PACAP; hence,

$$PACAP = L_{2} \times (0.2 \times a_{211} ) \times a_{212} \times a_{213} \times a_{214} /c,$$

where c is a constant of the same level of magnitude as L_2_, and defined in the equation to balance the level of magnitude of knowledge creation (R_22_).

Knowledge creation (R_22_) is an internal way of increasing a firm’s knowledge storage (Volberda et al. 2010). Knowledge storage (L_2_) is the foundation of knowledge creation (R_22_) and R&D investment (a_211_) is the main driving force. On the basis of our field interviews, it is assumed that 10 % of the firms’ R&D investments (a_211_) were used in knowledge creation (R_22_); therefore,

$$R_{22} = L_{2} \times (0.1 \times a_{211} )/c.$$

Outdated knowledge (R_23_) is a natural process by which old knowledge gradually becomes invalidated and can no longer be used in practice (Raman 2006). Logically, the rate at which knowledge becomes outdated (R_23_) depends on the amount of knowledge in the existing period controlled by both the quality of the knowledge itself and market demand selection. It is assumed that knowledge in the firm is large and evenly distributed among different types of knowledge, thus the rate at which knowledge becomes outdated (R_23_) can be simplified as the division of current knowledge storage (L_2_) by knowledge in the existing period (a_231_). Additionally, a_231_ is the production of the average knowledge-existing period (a_232_) obtained from the market research and the influential factor of market demand to the knowledge-existing period (a_233_), which is formulated as a table function of *market demand* (a_234_); thus,

$$R_{23} (t) = L_{2} /a_{231} .$$

$$a_{231} = a_{232} \times a_{233} ,\quad a_{233} = {\text{with-lookup(a}}_{ 2 3 4} ).$$

**Fig. 4:**
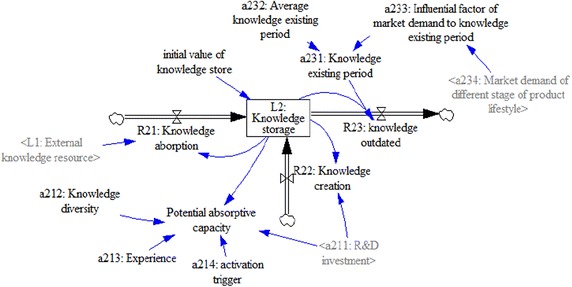
Separate stock-flow model of knowledge-storage subsystem

Figure 4 shows a stock-flow diagram of the knowledge-storage.

#### Technology-innovation-achievements subsystem

Technological innovation is the most important tangible outcome of absorptive capability and it is formed when enterprises transform and exploit existing knowledge (Cohen and Levinthal 1990; Zahra and George 2002). Technology-innovation achievements (L_3_) show the current technology level of a firm, which is changed by the inflow rate of technology innovation (R_31_) and the outflow of outdated technology (R_32_), with a specific initial value as follows:

$$\left\{ {\begin{array}{*{20}l} {\frac{{{\text{d}}L_{3} (t)}}{{{\text{d}}t}} = R_{31} (t) - R_{32} (t)} \hfill \\ {L_{3} (0) = {\text{ini}}\_L_{3} } \hfill \\ \end{array} } \right..$$

The technology-innovation rate (R_31_) is the process of applying a firm’s knowledge storage (L_2_) to technology development and commercial uses. The realized absorptive capacity (RACAP) makes the firm acceptant to transforming and exploiting external knowledge, which reflects its capacity to use the absorbed knowledge to improve innovation performance (Zahra and George 2002). Thus, RACAP positively affects the technology-innovation rate (R_31_). Moreover, R&D investment is also an important factor that influences technology innovation. According to field interviews in enterprises, 50 % of R&D investments (a_211_) go to internal technology innovation; hence,

$$R_{31} (t) = SQRT(RACAP \times L_{2} \times (0.5 \times a_{211} )/c).$$

Many factors influence RACAP and the first is PACAP (Zahra and George 2002). The second factor is R&D investment, and based on field interviews, most firms dedicate about 20 % of R&D investment to building RACAP. In addition, social integration mechanisms (a_311_), incentive systems (a_312_), and power relationships (a_313_) (Chang et al. 2013; Todorova and Durisin 2007) are positively related to RACAP; hence,

$$RACAP = SQRT(L_{2} \times (0.2 \times a_{211} ) \times a_{311} \times a_{312} \times a_{313} \times PACAP/c).$$

Outdated technology (R_32_) is a natural process and a consequence of market development, which means that a specific technology becomes less popular and gradually vanishes from the market. Technology here is considered as a mass stock, thus the rate of outdating is the result of the total amount of technology (L_3_) divided by technology in the existing period (a_321_). In turn, a_321_ is influenced by the market demand (a_234_) for the technology. R_32_ and a_321_ are then formulated as:

$$R_{32} (t) = L_{3} /a_{321} ;$$

$$a_{321} = {\text{with-lookup(a}}_{ 2 3 4} ).$$

Normally, there is a base R&D investment (a_322_) and a technology gap (a_111_) that determine the firm’s R&D investment (a_211_). In addition, there is an R&D period (a_323_) before the investment makes a practical contribution to the market. The formulation of R&D investment (a_211_) is:

$$a_{211} = (a_{322} + a_{111} )/a_{323} .$$

**Fig. 5:**
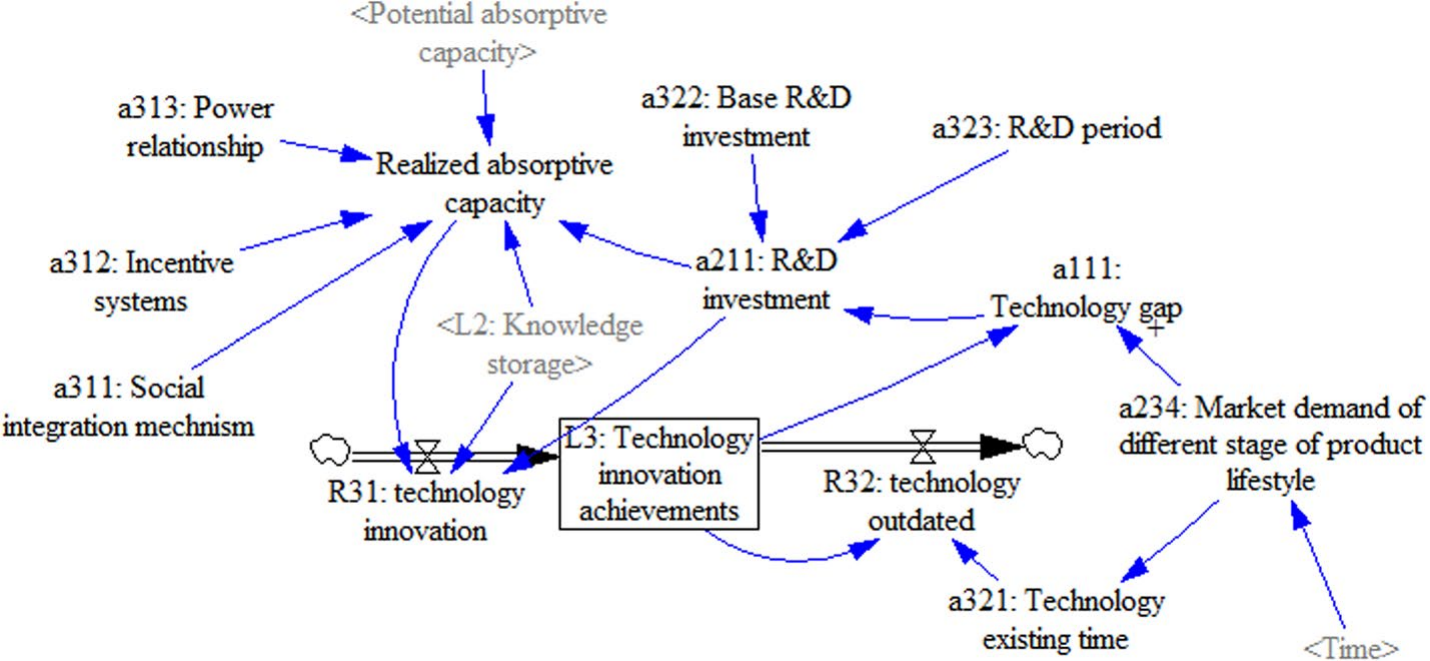
Separate stock-flow model of technology-innovation-achievements subsystem

Figure 5 shows the stock-flow diagram of the technology-innovation-achievements subsystem.

## Results and analysis

### Simulation results of the main variables

**Fig. 6:**
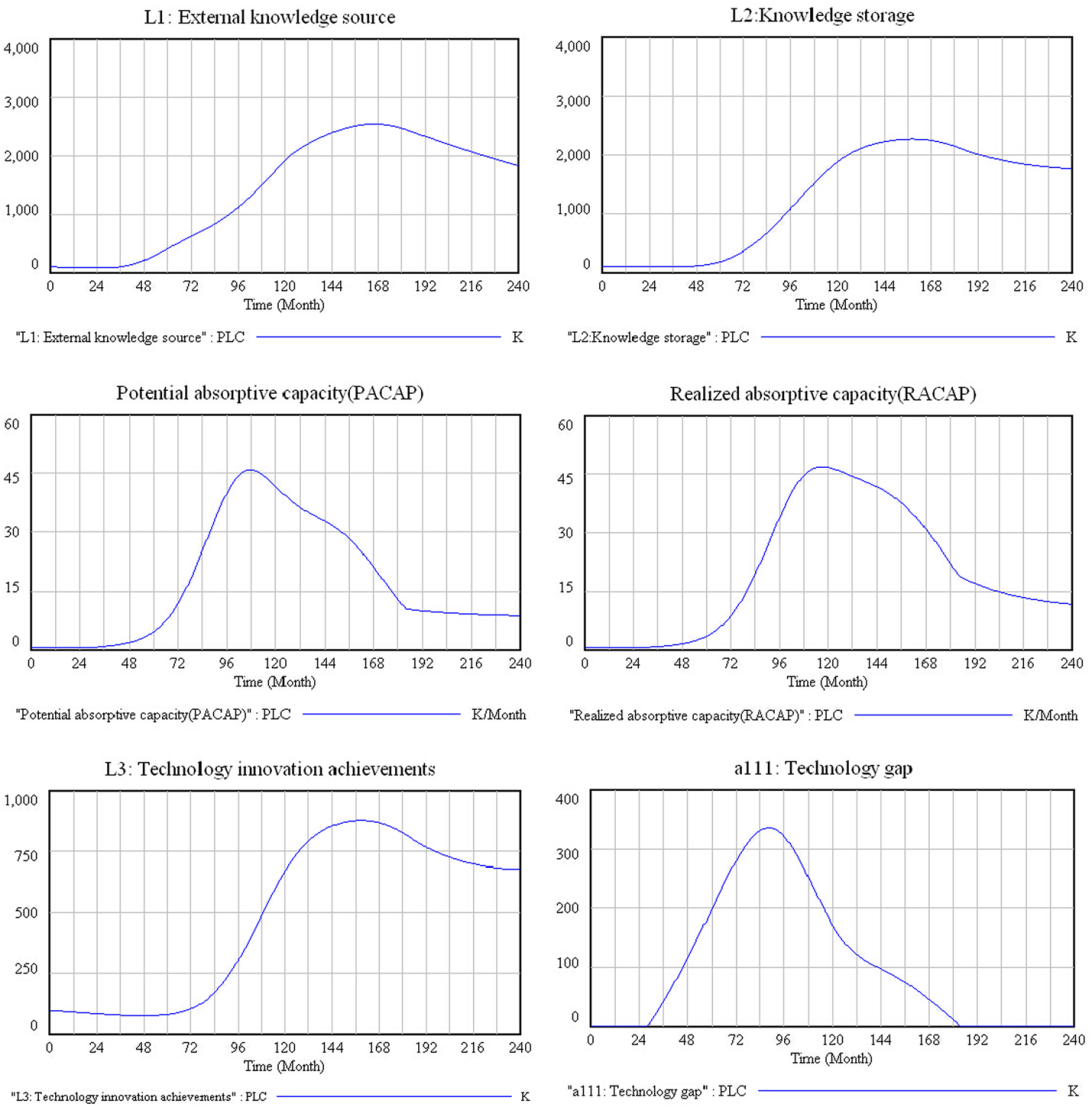
The tendencies of the main variables in the system

This paper adopts a long-term perspective (a short-term perspective is not possible) to investigate the dynamic nature of the new-product diffusion process (Cui et al. 2011b), and in particular, introducing PLC. In this study, a month is the simulation time unit, and the total simulation time consists of 240 months. The total simulation period is divided into four stages: introduction (from month 1 to month 60), growth (from month 61 to month 120), maturity (from month 121 to month 180), and decline (from month 181 to month 240). Figure 6 shows the simulation results for the six main variables under the different stages of PLC.

#### External knowledge source and knowledge storage

From the simulation results, we can see that the curves of the external knowledge source and knowledge storage are similar to the curve of the PLC, which means that the two variables are consistent with demand at different PLC stages. These changes are explained as follows. In the introduction stage, a product is put on the market, but market demand is not strong, and growth is slow (Golder and Tellis 2004). In this situation, the firm does not need to improve the overall technical performance of the product, thus the requirements of external knowledge are few. However, in the growth stage, the performance of the product in satisfying customer needs is crucial, and product modification may be necessary (Anderson and Zeithaml 1984). Therefore, firms need considerable knowledge in order to improve the product in this stage, and the importance of the firm’s external knowledge source increases far more rapidly. The objectives in the maturity stage are increasing efficiency, improving quality, and increasing product/market differentiation (Anderson and Zeithaml 1984). Firms still need significant knowledge to update the product in this stage, but the speed of knowledge demand is not as fast as in the growth stage. In the period of decline, because the product will be gradually phased out by the market (Golder and Tellis 2004), the firm’s need for an external knowledge source is drastically reduced.

Since firms’ knowledge storage mainly comes from external knowledge sources and internal knowledge creation (Cassiman and Veugelers 2006), the changing trend of the firms’ knowledge storage is similar to that of external knowledge sources.

#### Potential and realized absorptive capacities

According to the simulation results, the tendencies of potential absorptive capacity show changing trends in the different PLC stages. During the introduction stage, it increases slowly; in the growth stage, it increases rapidly and reaches a peak; in the mature stage, it begins to decline; and in the declining stage, it decreases slowly and stays at a low level. In the introduction stage, because the product has just been put on the market, a firm does not have to recognize and acquire a lot of external knowledge and its potential absorptive capacity is weak. However, in the growth stage, with the increased market demand, the firm needs to improve and update the product (Östlin et al. 2009). In order to meet the demand for product improvement, the firm needs to identify and acquire external knowledge quickly, thus potential absorptive capacity increases rapidly in this stage. In the mature stage, the market demand has been satisfied and product renewal is increasingly difficult compared with the growth stage. Thus, as the firm does not need to identify and acquire much external knowledge in this later stage, its potential absorptive capacity begins to decline. In the declining stage, the market demand for the product is drastically reduced until it is finally eliminated altogether (Golder and Tellis 2004) and the firm no longer has a need to identify and acquire external knowledge to complete the product. Therefore, potential absorptive capacity decreases to a stable level.

Realized absorptive capacity is based on the potential absorptive capacity, which involves external knowledge transformation and utilization (Zahra and George 2002). From our simulation results, we can see that the trend of realized absorptive capacity is similar to that of potential absorptive capacity.

#### Technology innovation achievements

As Fig. 6 shows, the technology innovation-achievement curves present various change tendencies in different PLC stages. During the introduction stage they are small, and increase sharply in the growth stage, while in the maturity stage the curves grow continually and reach a peak before beginning to decline and finally ultimately declining dramatically. From the overall tendency, the changes in a firm’s technology-innovation achievements are consistent with the PLC curve, which means that they are driven mainly by market demand. The result is consisted with previous studies that have demonstrated that market demand is the driving force of innovation (Cohen and Levinthal 1990; Lemma et al. 2015; Tempels and Van den Belt 2016).

#### Technology gap

According to the simulation results, the technology gap is zero in the period of introduction, but with the growth of market demand, the technology gap begins to emerge. In the growth stage, it increases rapidly and reaches its peak at month 84 before starting to decline. In the mature stage, it declines rapidly, and in the last stage, it finally it reaches zero. These changes are explained as follows. In the early period of introduction, the product has just been launched with the leading technology, and thus the technology gap is zero. As market demand grows, the technology gap gradually emerges and expands. In the growth stage, with the rapid increase in market demand, the technology-innovation achievements cannot meet the demand, thus the technology gap shows an expanding trend that reaches its peak. Meanwhile, the rapid growth of the new technology-innovation achievement comprises a part of the technology gap, which begins to diminish in the late period of the growth stage. In the maturity stage, the market demand has become saturated, while the firm’s technology-innovation achievements continue to increase, which means that the technology gap keeps reducing. In the declining stage, the market demand falls sharply, and the firm’s technology-innovation achievements can be completely satisfied by market demand, which means the technology gap has disappeared.

### Sensitivity analysis

To further test the model’s behaviors and the robustness, we conducted a sensitivity analysis of the model’s parameters. As pointed out by Cui et al. (2011b), rather than classify all parameters, we chose representative parameters and classified them into three groups related to three subsystems respectively. Based on the results of interviews and previous literature (Cohen and Levinthal 1990; Cui et al. 2011a; Fan 1995; Roberts et al. 2012; Zahra and George 2002), the parameters of time spent in founding an external knowledge network, an R&D period, and diverse knowledge are very important to the coevolution and effect of absorptive capacity, therefore we chose these three components as the parameters of the sensitivity analysis.

#### Time spent in founding an external knowledge network

**Fig. 7:**
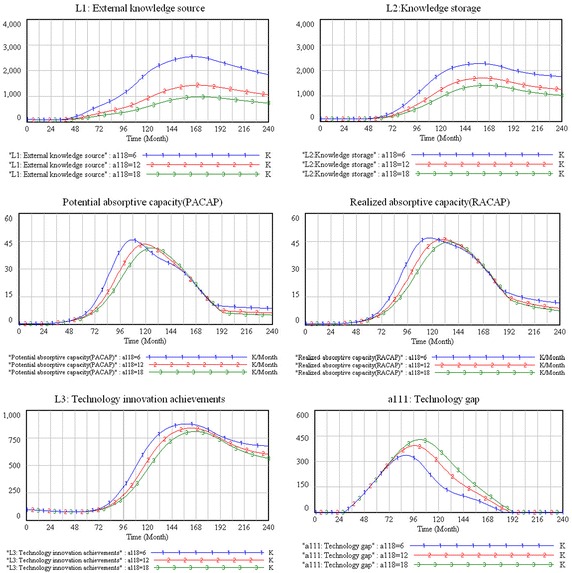
The sensitivity simulation results of time spent in founding an external knowledge network

An organization needs external knowledge to arrive at the right time (Cui et al. 2011a). In this study, the time spent in founding an external knowledge network is defined as a time delay from the recognition of a technology gap to taking action in order to find a suitable external knowledge source. This parameter is measured at 6, 12, and 18 months, respectively, and the results are shown in Fig. 7.

In Fig. 7, the simulation curves of all the main variables show almost no change under these three scenarios in the introduction stage, which means that the change of time spent in founding an external knowledge network does not affect the system’s behavior in this stage.

However, the system’s behavior is influenced by time spent in founding an external knowledge network in the growth stage. As shown in Fig. 7, external knowledge sources, knowledge storage, potential and realized absorptive capacities, and technology innovation achievements are increasing with shortening the time spent in founding an external knowledge network in this stage, while the technology gap is declining. Rink and Swan (1979) indicated that many competitors enter the market in the growth stage, which motivates enterprises to accelerate the speed of product updates and improvements to win the market share. Therefore, in a short period of time, firms establish an external knowledge network that can quickly increase the external knowledge sources and knowledge storage, which can improve the firm’s potential and realized absorptive capacities. In this way, the external knowledge network can improve firms’ technological-innovation performance and narrow the technology gap.

We can observe that the external knowledge source and knowledge storage of a firm both increase with a shortening of the time spent in founding an external knowledge network in the maturity stage. However, although the technology-innovation achievements still increase, the rate of increase slows down. One interesting finding is that, with the shortening of this parameter in this stage, the potential and realized absorptive capacities are reduced. We explain this interesting behavior as follows. Because the product has been greatly improved during the growth stage, the opportunities of product improvement are limited in the maturity stage. Therefore, the firm requires a longer time to identify, acquire, assimilate, and exploit external knowledge that support product improvement. As such, the firm must extend the time spent in founding an external knowledge network to improve its absorptive capacity and technological-innovation performance in this stage.

According to the simulation results, in the declining stage, external knowledge sources, knowledge storage, potential and realized absorptive capacities, and technology-innovation achievements are increased by shortening the time spent in founding the external knowledge network. One interesting finding is that although these variables are increased, the technology gap is always zero. As the product will be eliminated in the declining stage, the firm needs to make an innovative breakthrough for the product to survive. To achieve this goal, the firm has to shorten the time spent in founding an external knowledge network to support the achievement of a breakthrough innovation. Therefore, the external knowledge source, knowledge storage, and absorption capacity will be increased with the shortening of the time spent in founding an external knowledge network. However, because the technology level of the product can satisfy the market demand, the firm’s efforts in absorbing external knowledge and improving the product do not prevent a declining trend.

#### R&D period

**Fig. 8:**
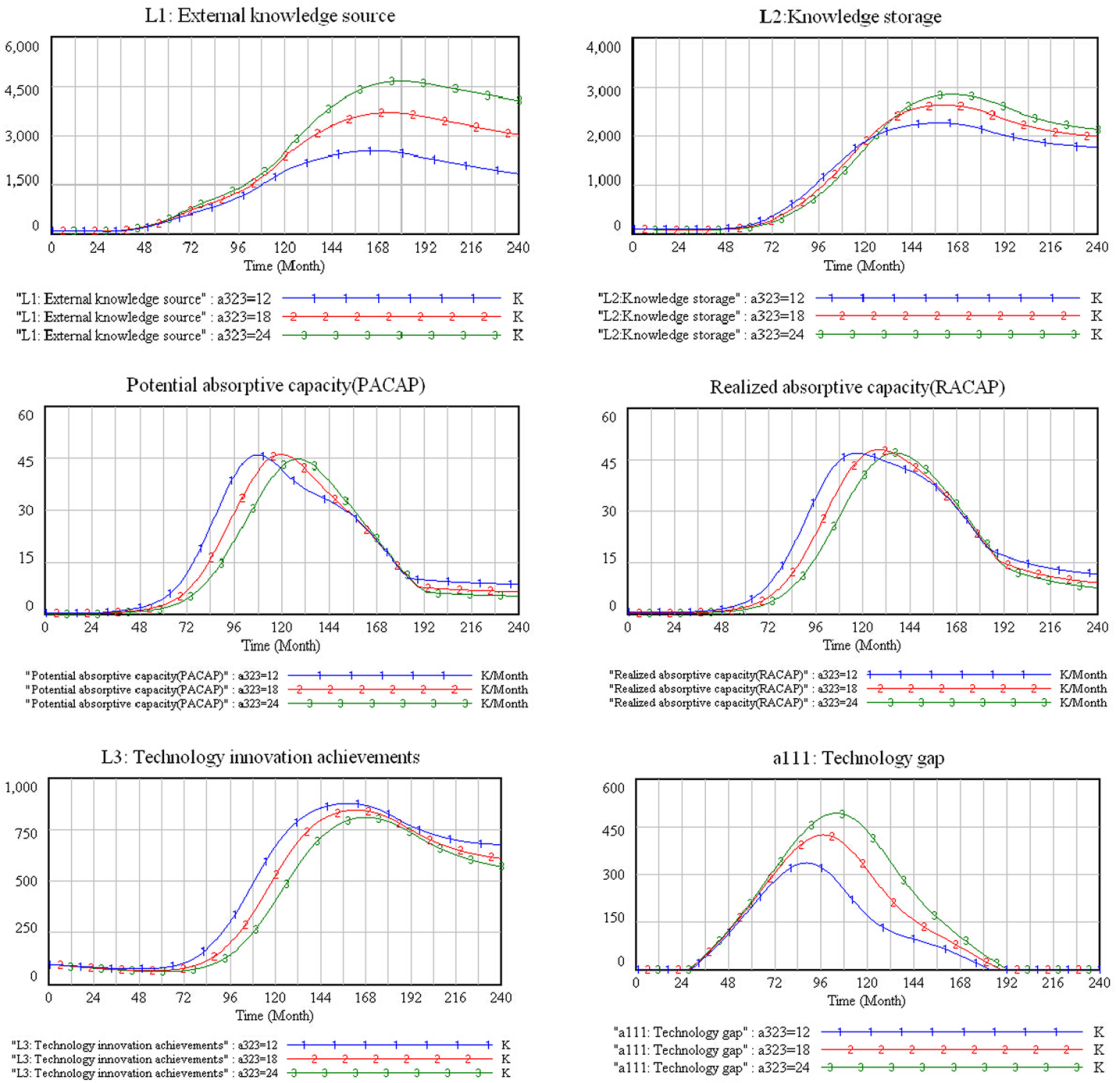
The sensitivity simulation results of R&D period

The R&D period is the time that it takes to develop new technology, which influences the firm’s innovation behavior significantly (Fan 1995). This period is key to R&D activities and innovation (Jin et al. 2014), which determines whether the firm can launch its innovations at the right time. In this study, the R&D period is given as 24, 18, and 12 months, and the simulation results are shown in Fig. 8.

According to Fig. 8, the simulation curves of all the main variables barely change during changes made during the R&D period in the introduction stage, which means that this period does not affect the system’s behavior in introduction stage.

However, the system behavior presents some obviously changes in the growth stage with changes made during the R&D period. Comparing the three scenarios shows that a shortened R&D period causes a reduction in the external knowledge source. In addition, although the knowledge storage, potential and realized absorptive capacities, and technology-innovation achievements are significantly increased, the technology gap is reduced significantly. These changes are interpreted as follows. First, the external knowledge networks that firms build decrease as the R&D period shortens, which leads to a decline in the firms’ external knowledge sources. Second, because product performance of a in satisfying customer needs is crucial, and since product modification may be necessary in the growth stage (Anderson and Zeithaml 1984), firms should cut down the R&D period in order to improve their products rapidly. This means that the firm has the pressure of identifying, acquiring, assimilating, and utilizing external knowledge in a shorter period of time, which leads to an improvement in the potential and realized absorptive capacities, as well as in the technological innovation performance.

According to the simulation results, the firms’ external knowledge source, knowledge storage, and potential and realized absorptive capacities are obviously reduced with shortening the R&D period in the maturity stage. We can also see that although the technology-innovation achievements increase with a shortening of the R&D period, the growth rate tends to change slowly. We explain the system behavior as follows. During the stage of maturity, the product becomes more and more standardized (Mahapatra et al. 2012)—even though it is already difficult to differentiate products through technical advantages (Kikuchi 2010). In other words, the opportunities of the product technology improvement are limited in this stage. Therefore, compared with the growth stage, it is not conducive for the firms to either increase their knowledge storage or identify, acquire, assimilate, and utilize external knowledge if the R&D period is reduced in the maturity stage.

Figure 8 shows the system behavior in the declining stage. We can see that with the shortening of the R&D period, the external knowledge source and knowledge storage are decreased, the potential and realized absorptive capacities, technology-innovation achievements are increased, and the technology gap is not affected. We explain the system behavior as follows. The market demand for the products is drastically reduced until it is eliminated in the declining stage (Golder and Tellis 2004). Hence, the firm no longer needs to spend money on building an external knowledge linkage and improving its knowledge storage. However, in order to survive in the market, the firm should try to assimilate and exploit the existing knowledge, which results in an increase in potential and realized absorptive capacities and technology innovation. In addition, due to the decreasing market demand for the product in this stage, the product’s existing technological level can meet market needs, so the technology gap is not affected by changes made in the R&D period.

#### Knowledge diversity

**Fig. 9:**
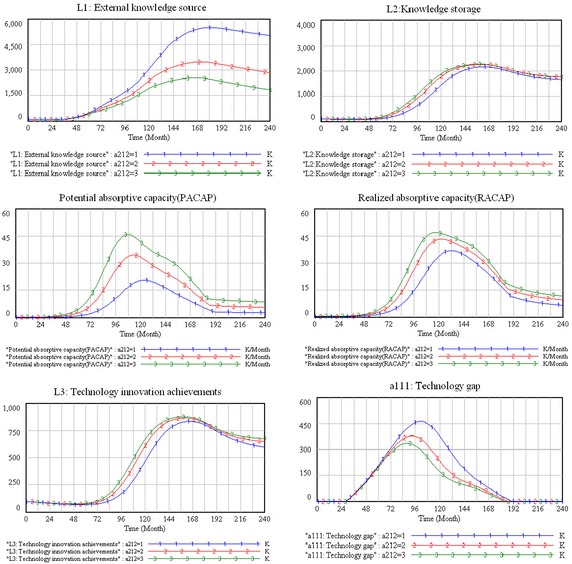
The sensitivity simulation results of knowledge diversity

Knowledge diversity is defined as the range of knowledge possessed by the organization with respect to the focal innovation (Fichman 2001; Roberts et al. 2012), which is one of most important factors that influences absorptive capacity (Cohen and Levinthal 1990; Roberts et al. 2012; Zahra and George 2002). In this study, the degree of this parameter is set at 1, 3, and 5, respectively, and the simulation results are shown in Fig. 9.

According to the simulation results, the main variables of the system remain almost constant during the introduction stage, which means that knowledge diversity has little effect on the behavior of the system in this stage.

However, the main variables change significantly under different scenarios in the growth stage. According to the simulation results, the external knowledge source is reduced with increasing knowledge diversity, and knowledge storage, potential, and realized absorptive capacities, and technology-innovation achievements all increase as the technology gap is reduced. We explain the system behavior as follows. Because the firm possesses a greater diversity of knowledge in the growth stage, which can meet the needs of product improvement, the firm will not build more external linkages. Karniouchina et al. (2011) argue that “When a new technique shows promise, innovators and early adopters expand its use and start perfecting its application, which lead to growth and increased effectiveness” (Karniouchina et al. 2011, p. 44), and thus the function and performance are the most important factors in the growth stage. To improve the function of a product, the firm needs to achieve rapid improvement, and thus a greater diversity of knowledge is conducive for identifying, acquiring, assimilating, and exploiting external knowledge effectively, thereby accumulating technology-innovation achievements and narrowing down the technology gap.

In the maturity stage, the simulation results show that, with the increase of knowledge diversity, the external knowledge source, the knowledge storage, and the technology gap are all reduced. An interesting finding is that although potential and realized absorptive capacities and technology innovation are growing in this stage, the growth rate slows with the increasing knowledge diversity. We explain these simulation results as follows. In the maturity stage, as the product gradually matures, the opportunities of product improvement are much more limited than in the growth stage, and therefore the firm needs more specialized knowledge to achieve a product-innovation breakthrough in this period. As Junkunc (2007) points out, specialized knowledge is focused on core business knowledge and is antecedent to an innovation breakthrough. In other words, in the maturity stage, the scattered knowledge diversity is not conducive to developing absorptive capacity and technology innovation.

During the decline stage, according to the results of the simulation, as knowledge diversity increases, the external knowledge source and knowledge storage decrease. Also, the potential and realized absorptive capacities and technology innovation achievements increase in this stage, while the technology gap is zero. The PLC declining stage for rapid innovators is marked by limited benefits (Nadeau and Casselman 2008). In other words, the market demand for products falls sharply, which means that products will soon be eliminated (Golder and Tellis 2004). In order ensure the product’s survival, the firm needs to put great effort into improving it. In this stage, increasing knowledge diversity makes it conducive for identifying, acquiring, assimilating, and exploiting external knowledge and achieving technology innovation. However, because market demand has declined, the firm’s existing technical level has been able to satisfy the market demands. Although the firm’s technology-innovation achievements are increased, the technology gap is still zero. Thus, the firm does not need to increase the knowledge diversity to improve the absorptive capacity and achieve technological innovation in the declining stage.

## Discussion and conclusions

### Theoretical contributions

Existing understanding of the dynamic nature of absorptive capacity has been confined to a comprehensive research framework, and few studies have revealed the dynamic process of absorptive capacity through a systematic view. This study develops a system dynamic model integrating important feedback loops among absorptive capacity, technological innovation, and product life cycle, and uncovers some interesting and new results. The specific contributions of the current theories in this field are set forth in the following paragraphs.

First, the past literature has primarily paid attention to the dynamic features of the absorptive capacity process, or the coevolution between absorptive capacity and the external environment, and less attention to developing an integrative framework. This study builds a system dynamic model that considers the dynamic process of absorptive capacity as well as one kind of external environment—the product life cycle. According to the simulation results, we find the following. (1) The PLC strongly affects the dynamic process of absorptive capacity. During the stages of growth and maturity, firms have a more active trend of valuing, acquiring, assimilating, transforming, and exploiting external knowledge than in the introduction and decline stages. (2) A firm’s potential and realized absorptive capacities reach their peak during the PLC growth stage. (3) The changing tendency of a firm’s technology-innovation achievements is consistent with the PLC curve, which means that technology-innovation achievements are driven mainly by market demand of PLC.

Second, a sensitivity analysis enriches our understanding of absorptive capacity and technological innovation with regard to the following aspects. (1) Time spent in funding an external knowledge network and R&D period influence absorptive capacity and innovation performance in different PLC stages and in different ways. During the stages of introduction and decline, Time spent in funding an external knowledge network and R&D period have almost no effect on absorptive capacity and innovation performance. However, if these factors are shortened in the growth stage, they are conducive to increases in absorptive capacity, as well as innovation performance. Finally, extending these factors during the maturity stage is conducive to increases in absorptive capacity and innovation performance. (2) Some scholars have found that knowledge diversity can increase absorptive capability and technology innovation achievements (Fichman 2001; Roberts et al. 2012), but our simulation results show that knowledge diversity has no effect in the introduction and declining stages of PLC, and too much knowledge diversity is not conducive to increasing absorptive capability and innovation performance in the maturity stage. It is only at the growth stage that increasing knowledge diversity has a positive effect.

Finally, for the first time, this paper attempts to build a system-dynamics model of absorptive capacity that provides a platform for further study. The model allows an examination of the dynamic feedback of absorptive capacity and technological innovation during the different PLC stages, and offers a research platform for studying the complex interactions of various external environments (e.g., fluctuated market, environment jolt) that impact absorptive capacity and innovation.

### Managerial contributions

For the purpose of improving a firm’s absorptive capacity and technological innovation performance, the firm needs to develop a strategy that matches the market demand. According to the sensitivity simulation results of this study, management policies are put forward at the firm at different PLC stages, which are presented in Table 1.

**Table 1:** The management policy for firm adopting in different stages of PLC

	Introduction stage	Growth stage	Maturity stage	Declining stage
Time spent in founding an external knowledge network	Without consideration	Shorten	Extend	Without consideration
R&D period	Without consideration	Shorten	Extend	Without consideration
Knowledge diversity	Without consideration	Increase	Decrease	Without consideration

In the introduction stage, the firm does not need to take the factors of time spent in founding an external knowledge network, the R&D period, and knowledge diversity into consideration because the product has just been brought into the market and demand is low. The firm’s main task at this stage is to induce customers to accept the product and increase the market demand.

However, due to increasing demand, competitors appear in the market in the growth stage, so the firm must improve the product in a short time by absorbing external knowledge in order to retain its competitive advantage. To achieve such rapid innovation, the firm needs to shorten the time spent in founding an external knowledge network, which can help the firm to quickly obtain more external knowledge. The firm also needs to shorten its R&D period, which can be conducive to quickly assimilating and exploiting external knowledge and achieving product innovation. Meanwhile, the firm should constantly increase its knowledge diversity, which is conducive to identifying, acquiring, assimilating, and exploiting external knowledge.

In the maturity stage, because the market demand for the product reaches its maximum, the opportunities of product update and improvement is greatly reduced. Thus, in order to carry out additional product innovation at this stage, the firm needs to spend more time identifying and founding an external knowledge network to effectively compete with new market entrants. In addition, the firm also needs to extend the R&D period to facilitate more comprehensive and deeper technological innovation activities, while also reducing knowledge diversity and increasing specialized knowledge to realize the necessary technological innovation.

In the declining stage, the market demand for the product continues to decrease, and the product is probably going to pass out of the market soon. Our simulation results show that in this stage, the technology gap is zero—i.e., the product’s technological level has been fully able to meet the market demand. This also means that in this stage, the firm’s endeavors with regard to product innovation cannot change the trend that will result in the product’s eventual elimination. Therefore, at this stage, the firm does not need to consider how to control the time involved in founding an external knowledge network, the R&D period, or knowledge diversity.

### Limitations

This paper has some limitations. First, this paper unified most variable dimensions in the model by the knowledge unit (K) in order to simplify the model and facilitate study. Although this approach conforms to the basic principle of system-dynamics modeling and can reasonably explain the variables in theory, this processing method does not represent actual situations of enterprises. Second, the parameter assignment of each equation in the model is based on the interview results, which may not be rigorous enough, so in further studies the questionnaire could be used to collect more rigorous data to make the model more closely representative of reality. Third, the survey’s sample is confined to firms in China, which may limit the generalization of the findings to other country.

## Appendix

See Table 2.

**Table 2:** Parameters in the model

Variables	Unit (value/value range)
L_1_: External knowledge source	K
Initial value of L_1_	K (0)
R_11_: Inflow rate of establishing external knowledge	K/month
a_111_: Technology gap	K
a_112_: Knowledge gap	K
a_113_: Average external knowledge level	K
a_114_: Influential factor of knowledge gap	1 (0–3)
a_115_: Organizational difference	1 (1–5)
a_116_: Physical distance	1 (1–5)
a_117_: Trust level	1 (1–5)
a_118_: Time spent in founding an external knowledge network	Month (3–36)
R_12_: Outflow rate of knowledge absorption	K/month
L_2_: Knowledge storage	K
Initial value of knowledge storage	K (100)
R_21_: Inflow rate of knowledge absorption	K/month
a_211_: R&D investment	K/month
a_212_: Knowledge diversity	1 (1–5)
a_213_: Experience	1 (1–5)
a_214_: Activation trigger	1 (1–5)
R_22_: Inflow rate of knowledge creation	K/month
R_23_: Outflow rate of outdated knowledge	K/month
a_231_: Knowledge existing period	Month
a_232_: Average knowledge existing period	Month
a_233_: Influential factor of market demand to knowledge existing period	1 (0–3)
a_234_: Market demand of different stage of product life cycle	K
L_3_: Technology innovation achievements	K
Initial value of technology innovation achievements	K (100)
R_31_: Inflow rate of technology innovation	K/month
a_311_: Social integration mechanisms	1 (1–5)
a_312_: Incentive systems	1 (1–5)
a_313_: Power relationship	1 (1–5)
R_32_: Outflow rate of outdated technology	K/month
a_321_: Technology existing time	Month
a_322_: Base R&D investment	K
a_323_: R&D period	Month (6–60)
Potential absorptive capacity (PACAP)	K/month
Realized absorptive capacity (RACAP)	K/month
